# Pancreatic Cancer Risk in Patients With Low-Risk Cystic Lesions

**DOI:** 10.1001/jamanetworkopen.2026.13808

**Published:** 2026-05-20

**Authors:** Arya Haj Mirzaian, Nooshin Abbasi, Avinash R. Kambadakone, Ronilda Lacson, Yasmin G. Hernandez-Barco, David X. Jin, Kunal Jajoo, David W. Bates, Ramin Khorasani

**Affiliations:** 1 Center for Evidence-Based Imaging, Department of Radiology, Brigham and Women’s Hospital, Harvard Medical School, Boston, Massachusetts; 2 Department of Radiology, Brigham and Women’s Hospital, Harvard Medical School, Boston, Massachusetts; 3 Department of Radiology, Massachusetts General Hospital, Harvard Medical School, Boston; 4 Department of Gastroenterology, Massachusetts General Hospital, Harvard Medical School, Boston; 5 Center for Pancreatic Disease, Division of Gastroenterology, Hepatology, and Endoscopy, Department of Medicine, Brigham and Women’s Hospital, Harvard Medical School, Boston, Massachusetts; 6 Division of Gastroenterology, Hepatology and Endoscopy, Brigham and Women’s Hospital, Harvard Medical School, Boston, Massachusetts; 7 Division of General Internal Medicine and Primary Care, Department of Medicine, Brigham and Women’s Hospital, Harvard Medical School, Boston, Massachusetts; 8 Department of Healthcare Policy and Management, Harvard T.H. Chan School of Public Health, Boston, Massachusetts

## Abstract

**Importance:**

Low-risk pancreatic cystic lesions (PCLs) represent common incidental findings with potential for malignant transformation, warranting long-term surveillance. However, data on their long-term cancer risk are limited, leading to inconsistency in current surveillance strategies.

**Objective:**

To determine the long-term incidence of pancreatic cancer among patients with low-risk PCLs, and to identify baseline clinical and imaging factors associated with cancer development.

**Design, Setting, and Participants:**

This cohort study was conducted among adults (aged ≥18 years) who underwent contrast-enhanced abdominal computed tomography (CT) or magnetic resonance imaging (MRI) between January 1, 2009, and December 31, 2021, at a large, multisite health care system in Massachusetts. MRI or CT scans were screened for PCLs without high-risk stigmata, worrisome features, or coexisting pancreatic mass. Eligible patients with low-risk PCLs were longitudinally followed up. Data analysis was performed from February 2024 to May 2025.

**Exposures:**

Baseline demographic, clinical, and imaging characteristics of patients with low-risk PCLs.

**Main Outcomes and Measures:**

The outcome was incidence of pancreatic cancer (defined as pathologically confirmed adenocarcinoma, invasive mucinous neoplasms, or high-grade dysplasia). Censoring was defined as absence of pancreatic cancer on imaging follow-up for more than 1 year after the initial examination documenting the PCL. Multivariable cause-specific Cox proportional hazards regression models assessed the associations between baseline factors and incident pancreatic cancer, accounting for death as a competing risk. Associations were reported as hazard ratios (HRs). Risk stratification performance was assessed using Harrell C-statistic and net reclassification index (NRI).

**Results:**

Among 499 631 patients reviewed, 6064 with low-risk PCLs were identified and included in the analytic sample, contributing 20 145 person-years of follow-up. Patients had a mean (SD) age at diagnosis of 65.9 (12.3) years and included 3612 females (59.6%). Of the 6064 patients, 38 (0.6%) developed pancreatic cancer, with an incidence rate of 1.89 (95% CI, 1.29-2.49) cases per 1000 person-years, which was higher than the previously reported general population rate of 0.14 cases per 1000 person-years. Twenty-six of 38 patients (68.4%) had cancer that arose from the cyst site, while 12 (31.6%) developed cancer from a different region of the pancreas. Ten patients (26.3%) were diagnosed more than 5 years after initial PCL detection. In multivariable analysis, larger cyst size (HR, 2.24; 95% CI, 1.45-3.48), main pancreatic duct ectasia (HR, 2.84; 95% CI, 1.18-6.84), and older age (HR, 1.04; 95% CI, 1.01-1.07) were associated with cancer. Adding age to a cyst size–based risk stratification model improved estimation of pancreatic cancer risk (NRI, 0.20 [95% CI, 0.03-0.37]; change in C-statistic, 0.14 [95% CI, 0.07-0.22]).

**Conclusions and Relevance:**

This cohort study found that low-risk PCLs were associated with a sustained long-term pancreatic cancer risk and were best stratified by combining clinical and imaging factors. Longer than a 5-year follow-up of low-risk PCLs may be warranted to reduce missed or delayed diagnosis of pancreatic cancer.

## Key Points

**Question:**

Which baseline factors are associated with long-term pancreatic cancer incidence among patients with low-risk pancreatic cystic lesions (PCLs)?

**Findings:**

In this cohort study of 6064 patients with low-risk PCLs followed up for 20 145 person-years, pancreatic cancer incidence was 1.89 cases per 1000 person-years, approximately 14-fold higher than the general population rate. Approximately one-third of cancers arose outside the cyst site; 26.3% occurred more than 5 years after PCL detection.

**Meaning:**

These findings suggest that low-risk PCLs carry a nonnegligible long-term risk of cancer, supporting the need for personalized surveillance strategies.

## Teaser

This cohort study evaluates incidence of pancreatic cancer and the clinical and imaging factors associated with cancer development among adults with low-risk pancreatic cystic lesions.

## Introduction

Pancreatic cancer is the most lethal malignant neoplasm, with a 5-year survival rate of less than 15%.^1^ This poor outcome is largely due to late-stage diagnosis; when the disease is detected early, the survival rate can rise to approximately 80%.^2,3^ Therefore, early detection of pancreatic cancer or its precursors is critical and remains an important clinical goal.

Pancreatic cystic lesions (PCLs) are among the most recognized precursors of pancreatic cancer, are present in up to 50% of the general population, and have a rising prevalence due to increased use and quality of imaging as well as population aging.^4,5,6,7^ PCL management is challenging due to PCLs’ diverse biological behavior, encompassing a spectrum of benign to premalignant and malignant states.^5,8^ For high- and intermediate-risk PCLs, defined by the presence of high-risk stigmata or worrisome features, guidelines typically recommend diagnostic intervention with endoscopic ultrasonography (EUS)–guided fine needle aspiration (FNA) or surgical resection.^9,10,11,12,13,14,15^ However, the greater clinical challenge lies in the management of low-risk PCLs, which constitute the majority of PCLs.^8^ In these cases, balancing imaging surveillance and intervention becomes challenging, as unnecessary procedures may cause harm without providing benefit, while inadequate monitoring could lead to missed malignant neoplasms.^16,17,18^

Despite the high prevalence of low-risk PCLs, their exact risk of pancreatic cancer remains unclear due to the limited availability of longitudinal studies.^8,19^ Many studies assessing PCL risk are subject to verification bias, as they primarily include patients who underwent resection, excluding the majority of PCLs that are managed conservatively.^9,10,11,12,13,14,15^ Current management of low-risk PCLs is heterogeneous and relies heavily on expert opinion with a low strength of supporting evidence.^9,20,21^ These factors are associated with uncertainty in clinical management. For instance, studies have demonstrated a 2.8-fold variation among radiologists in recommending additional imaging for PCL management.^22,23^

Given the scope of this clinical challenge and the limitations of current practice, building an evidence-based approach to managing low-risk PCLs is essential. Our multisite health care network, with more than 3 million imaging encounters annually, enabled us to make a comprehensive retrospective assessment of a large group of patients with low-risk PCLs. Our study aimed to determine the long-term incidence of pancreatic cancer among patients with low-risk PCLs and to identify baseline clinical and imaging factors associated with cancer development.

## Methods

### Study Design and Participants

This Health Insurance Portability and Accountability Act–compliant retrospective cohort study was conducted at a large multisite medical center in Massachusetts, including 3 tertiary or quaternary care hospitals and 7 community hospitals with a total of 3853 beds. The Mass General Brigham Institutional Review Board approved the study and waived the informed consent requirement because retrospective analysis of existing data posed minimal risk to participants. We followed the Strengthening the Reporting of Observational Studies in Epidemiology (STROBE) reporting guideline.^24^

**Figure 1.**
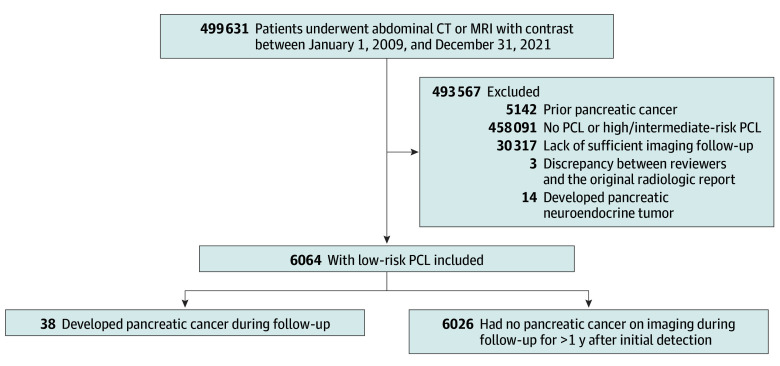
Flowchart of Patient Participation CT indicates computed tomography; MRI, magnetic resonance imaging; PCL, pancreatic cystic lesion.

All adult patients (aged ≥18 years) who underwent contrast-enhanced abdominal computed tomography (CT) or magnetic resonance imaging (MRI) for any indication between January 1, 2009, and December 31, 2021, were identified (Figure 1). Eligible patients’ imaging studies were required to include late arterial and/or portal venous phases because these are optimal for pancreatic assessment. Patients whose radiologic report documented PCL presence were identified using a validated machine-learning algorithm incorporating natural language processing (NLP).^25^

After identifying patients with PCLs, an index examination was identified as the first abdominal CT or MRI documenting a PCL. If a patient had an older abdominal CT or MRI (prior to the study period), then the oldest imaging record was found (by A.H.M.) and assigned as the index examination to maximize follow-up duration. Next, the medical records and index examination reports were manually reviewed (by A.H.M.) to identify patients with low-risk PCLs, while applying the following exclusion criteria: (1) presence of high-risk stigmata per standard guidelines,^8,26^ defined as biliary obstruction, main pancreatic duct (MPD) larger than 10 mm, solid enhancing mass or mural nodules of 5 mm or larger, or obstructive jaundice; (2) presence of worrisome features,^8,26^ including PCL size larger than 3 cm, MPD of 5 to 10 mm, MPD stricture, enhancing mural nodules smaller than 5 mm, enhancing septations, thickened or enhancing cyst wall, acute pancreatitis (based on clinical or imaging results; patients with prior resolved pancreatitis were not excluded), or abdominal lymphadenopathy; (3) concurrent suspicious solid pancreatic mass; and (4) history of pancreatic malignant neoplasm (ie, pancreatic adenocarcinoma, invasive mucinous neoplasms, or at least high-grade dysplasia) prior to the index examination.

### Outcome

The study outcome was pancreatic cancer incidence any time after initial identification of a low-risk PCL. Pancreatic cancer was defined as a pathologically confirmed adenocarcinoma, invasive mucinous neoplasms, or at least high-grade dysplasia, reflecting advanced neoplasia.^8,27^ Censoring was defined as absence of pancreatic cancer on follow-up imaging (contrast-enhanced CT, MRI, or EUS) during the study period; patients were required to have at least 1 year of imaging follow-up. Patients were excluded if they lacked sufficient follow-up or if they developed pancreatic neuroendocrine tumors; these tumors are outside the primary scope of PCL surveillance and can often be managed conservatively.^28^

### Data Collection

The radiologic report text of the index examination and patient demographic and clinical data (at the time of the index examination) were retrieved from the Mass General Brigham Enterprise Data Warehouse. Pancreatic cancer diagnoses were retrieved from institutional cancer registry databases, which provided information on the tissue confirmation date and the histopathologic subtype.

PCL-specific data were manually extracted from the text of radiologic reports (by A.H.M. and N.A., both with 6 years of radiology research experience). These data included the maximum size of the largest PCL (for patients with multiple PCLs, the largest lesion was recorded), presence of multiple PCLs, MPD ectasia (diameter of 3-5 mm), PCL location (head, uncinate process, neck, body, or tail; for multiple PCLs in different locations, all locations were recorded), chronic pancreatitis,^29^ pancreatic divisum, and imaging features suggestive of serous cystadenoma,^30^ pseudocysts, or fluid collections. Structured patient-specific data were retrieved from the data warehouse, including age, self-reported sex, self-reported race and ethnicity (Asian, Black, Hispanic White and non-Hispanic White, and other [American Indian or Alaska Native, Native Hawaiian or Other Pacific Islander, and multiracial]), family history of pancreatic adenocarcinoma (first- or second-degree relatives), medical history (diabetes and obesity or overweight), smoking status (ever smoked), and alcohol use (mean units per week during the study period). Race and ethnicity were included in this analysis because they may be associated with differences in the incidence of pancreatic cancer.

### Quality Assurance

Radiologic reports from a random sample of 100 patients were independently reviewed (by A.H.M. and N.A.) to assess agreement between manually extracted and NLP-derived data for identifying PCLs in radiologic reports. In addition, all CT and MR scans of patients with low-risk PCLs who subsequently developed pancreatic cancer, as well as images from a random sample of 120 patients who did not develop cancer, were reviewed (by A.H.M., who has 4 years of experience in interpreting abdominal imaging).

### Statistical Analysis

Baseline characteristics were summarized using descriptive statistics. A Z-test was used to compare the observed pancreatic cancer incidence rate with a previously reported population incidence.

Univariable and multivariable cause-specific Cox proportional hazards regression models were used to assess associations between baseline variables and pancreatic cancer incidence, accounting for death as a competing risk (eMethods in Supplement 1). Cumulative incidence functions were estimated using the Aalen-Johansen method. Variables included in the multivariable model were selected based on clinical relevance, statistical findings in univariable analysis, and absence of collinearity (variance inflation factor of >10).^31^ The associations were reported as hazard ratios (HRs) with corresponding 95% CIs. Patients were analyzed according to baseline characteristics measured at the index examination. Missing baseline covariate data were addressed using multivariate imputation by chained equations. Cohen κ statistic was calculated to assess NLP accuracy for identifying PCLs.

To evaluate the incremental value of adding factors identified in the multivariable model to the cyst size–based risk stratification, we assessed changes in discrimination and reclassification. Discrimination was assessed using Harrell C-statistic with bootstrap-estimated differences between models. Improvement in risk classification was assessed using the net reclassification index (NRI).^32,33^ Selected continuous variables were dichotomized using area under the curve analysis, with the optimal cutoff determined by the Youden index.

All analyses were carried out from February 2024 to May 2025 using Python, version 3.10 (Python Software Foundation). All hypothesis tests were 2-sided, with a significance threshold set at *P* < .05.

## Results

### Baseline Characteristics

Among 499 631 patients reviewed, 6064 (1.2%) with low-risk PCLs were identified, contributing 20 145 person-years of follow-up (Figure 1). These patients had a mean (SD) age at diagnosis of 65.9 (12.3) years and included 3612 females (59.6%) and 2451 males (40.4%) (Table 1). Of these patients, 222 (3.7%) identified as Asian, 225 (3.7%) as Black, and 5024 (82.8%) as Hispanic or non-Hispanic White individuals, with 227 (3.7%) reporting other race, including American Indian or Alaska Native, Native Hawaiian or Other Pacific Islander, and multiracial. At baseline, 4188 patients (73.3%) had subcentimeter PCLs. The mean (SD) proportion of missing data across all variables was 0.03% (0.06%) (eTable 1 in Supplement 1).

**Table 1.** Baseline Characteristics of Patients With Low-Risk PCLs

Characteristic	Patients, No. (%) (N = 6064)
Demographic or clinical	
Age, mean (SD), y	65.9 (12.3)
Sex^a^	
Male	2451 (40.4)
Female	3612 (59.6)
Race and ethnicity^a^^,^^b^	
Asian	222 (3.7)
Black	225 (3.7)
White^c^	5024 (82.8)
Other^d^	227 (3.7)
Diabetes	340 (5.6)
Obesity or overweight	158 (2.6)
Family history of pancreatic adenocarcinoma	114 (1.9)
Ever smoked	2886 (47.6)
Alcohol use, mean (SD), units per wk	3.1 (16.2)
Imaging features	
Imaging modality^a^	
MRI	3684 (60.8)
CT	2368 (39.1)
PCL size: maximum diameter of the largest cyst^a^	
<1 cm	4188 (73.3)
1 to <2 cm	1261 (22.1)
2 to <3 cm	268 (4.7)
PCL location in each patient^a^	
Head	1398 (23.1)
Uncinate process	902 (14.9)
Neck	578 (9.5)
Body	1740 (28.7)
Tail	1538 (25.4)
Multiple PCLs	2403 (39.6)
MPD ectasia^e^	277 (4.6)
Imaging features of chronic pancreatitis	127 (2.1)
Pancreatic divisum	210 (3.5)
Suggestive of pseudocyst	259 (4.3)
Suggestive of fluid collection	405 (6.7)

Abbreviations: CT, computed tomography; MPD, main pancreatic duct; MRI, magnetic resonance imaging; PCL, pancreatic cystic lesion.

^a^ Percentages and counts may not sum to 100% and 6064, respectively, because of missing data or overlapping categories.

^b^ Race and ethnicity were self-identified by patients and obtained from the data warehouse.

^c^ Includes Hispanic White and non-Hispanic White.

^d^ Other includes American Indian or Alaska Native, Native Hawaiian or Other Pacific Islander, and multiracial.

^e^ Defined as 3-mm to 5-mm MPD diameter.

### Data Validation and Quality Assurance

There was a high level of agreement between manually extracted and NLP-derived data for identifying PCLs (κ = 0.99). On manual imaging review, 98.1% (155 of 158) of patients showed no discrepancy between reviewer interpretation and the original radiologic reports. Three cases (1.9%) had additional findings on manual review: 2 with MPD abrupt cutoff that was not originally reported and 1 with a 7-mm MPD that was originally reported as 4 mm. These patients were excluded from the analysis, as they were not truly low risk. Therefore, imaging review of all included patients who developed pancreatic cancer confirmed the presence of low-risk PCLs.

### Pancreatic Cancer Incidence

**Figure 2.**
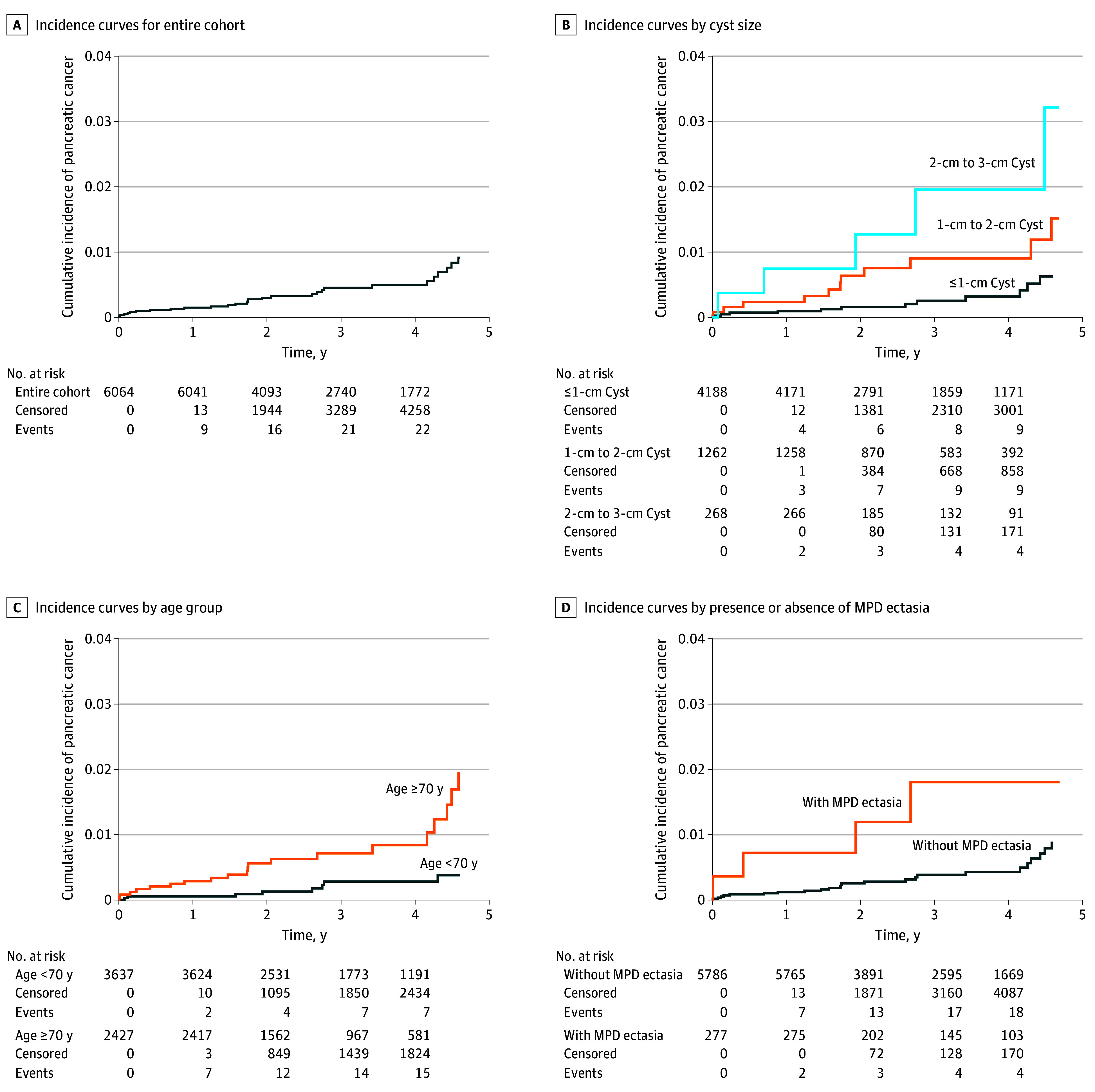
Cumulative Incidence Curves for the Entire Cohort and by Subgroups of Patients With Low-Risk Pancreatic Cystic Lesions Curves were truncated when less than 20% of the cohort remained under follow-up. MPD indicates main pancreatic duct.

The mean (IQR) duration of follow-up was 3.3 (1.7-4.3) years. Among 6064 patients, 2741 (45.2%) were followed up for more than 3 years and 1013 (16.7%) were followed up for more than 5 years. During follow-up, 38 patients (0.6%) developed pancreatic cancer, with an incidence rate of 1.89 (95% CI, 1.29-2.49) cases per 1000 person-years, which was significantly higher than the previously reported pancreatic cancer incidence in the previously reported general population of 0.14 cases per 1000 person-years (*P* < .001).^34,35,36^ During follow-up, 22 participants (0.4%) died without pancreatic cancer; these deaths were treated as competing events. Among the 38 patients with pancreatic cancer, 9 (23.7%) were diagnosed within 1 year from the index examination, 19 (50.0%) were diagnosed between 1 and 5 years, and 10 (26.3%) were diagnosed after 5 years (Figure 2; eTable 2 in Supplement 1). Thirty-seven (97.4%) had pathologically confirmed adenocarcinoma, and 1 (2.6%) had high-grade dysplasia. Twenty-six patients (68.4%) developed cancer originating from the PCL site, while 12 (31.6%) developed cancer in a different region of the pancreas. Cancer from larger PCLs (>1 cm) more often originated from the cyst site, whereas cancer from subcentimeter PCLs more often originated elsewhere in the pancreas (eTable 2 in Supplement 1).

### Factors Associated With Pancreatic Cancer Incidence

Univariable Cox proportional hazards regression analysis identified PCL size (<1, 1-2, or 2-3 cm)^8^ (HR, 2.51 [95% CI, 1.64-3.54] per category increase) and MPD ectasia (HR, 3.31; 95% CI, 1.38-7.96) as significant imaging factors associated with pancreatic cancer development (Table 2). Among clinical variables, older age at the time of index examination (HR, 1.05; 95% CI, 1.02-1.08) and male sex (female [vs male reference group]: HR, 0.49; 95% CI, 0.26-0.95) were associated with a higher pancreatic cancer incidence (Table 2).

**Table 2.** Association Between Baseline Variables and Incidence of Pancreatic Cancer in Univariable and Multivariable Cox Proportional Hazards Regression Models

Variable	Incidence of pancreatic cancer			
	Univariable model		Multivariable model	
	HR (95% CI)	*P* value	HR (95% CI)	*P* value
Age, y	1.05 (1.02-1.08)	.002	1.04 (1.01-1.07)	.02
Sex				
Male	1 [Reference]	NA	1 [Reference]	NA
Female	0.49 (0.26-0.95)	.03	0.59 (0.31-1.13)	.11
PCL size: maximum diameter of the largest cyst				
Per category increase	2.51 (1.64-3.54)	<.001	2.24 (1.45-3.48)	<.001
1-2 vs <1 cm	1.25 (0.62-2.54)	.54	1.53 (0.71-3.29)	.28
2-3 vs <1 cm	5.68 (2.67-12.06)	<.001	5.61 (2.47-12.75)	<.001
MPD ectasia^a^	3.31 (1.38-7.96)	.007	2.84 (1.18-6.84)	.02
Modality				
CT	1 [Reference]	NA	NA	NA
MRI	0.62 (0.33-1.20)	.16	NA	NA
Race and ethnicity, categorical	0.80 (0.52-1.23)	.31	NA	NA
Diabetes	0.36 (0.05-2.60)	.31	NA	NA
Obesity or overweight	NC	.99	NA	NA
Family history of pancreatic adenocarcinoma	0.89 (0.12-6.51)	.90	NA	NA
Ever smoked	1.16 (0.61-2.22)	.64	NA	NA
Alcohol use, mean units per wk	0.98 (0.92-1.04)	.45	NA	NA
PCL location (all PCLs in each patient)				
Head	1.22 (0.60-2.46)	.59	NA	NA
Uncinate process	0.55 (0.19-1.55)	.26	NA	NA
Neck	0.71 (0.22-2.32)	.57	NA	NA
Body	1.02 (0.51-2.03)	.96	NA	NA
Tail	1.19 (0.60-2.36)	.63	NA	NA
Multiple PCLs	1.45 (0.76-2.76)	.26	NA	NA
Imaging features of chronic pancreatitis	2.38 (0.57-9.89)	.23	NA	NA
Pancreatic divisum	1.73 (0.42-7.18)	.45	NA	NA
Suggestive of pseudocyst	1.05 (0.25-4.38)	.95	NA	NA
Suggestive of fluid collection	1.37 (0.49-3.88)	.55	NA	NA
Suggestive of serous cystadenoma	1.54 (0.78-3.04)	.21	NA	NA

Abbreviations: CT, computed tomography; HR, hazard ratio; MPD, main pancreatic duct; MRI, magnetic resonance imaging; NA, not applicable; NC, nonconvergence of model due to low event rate; PCL, pancreatic cystic lesion.

^a^ Defined as 3-mm to 5-mm MPD diameter.

In the multivariable model, PCL size (HR, 2.24 [95% CI, 1.45-3.48] per category increase), MPD ectasia (HR, 2.84; 95% CI, 1.18-6.84), and age (HR, 1.04; 95% CI, 1.01-1.07) remained significantly associated with pancreatic cancer incidence (Table 2). An age threshold of 70 years or older, derived using the Youden index, was identified as a descriptive reference point. Cumulative incidence analysis demonstrated a significantly higher risk of pancreatic cancer in patients with larger PCLs, MPD ectasia, and older age (Figure 2).

### Exploratory Analyses: Incremental Value of Adding Age and/or MPD Ectasia to Cyst Size–Based Risk Stratification

Compared with a cyst size–only model (≥2 cm; C-statistic, 0.57 [95% CI, 0.51-0.64]), adding age (≥70 years) significantly increased discrimination (C-statistic, 0.71 [95% CI, 0.64-0.78]; change in C-statistic, 0.14 [95% CI, 0.07-0.22]; *P* < .001) (eTable 3 in Supplement 1). Adding MPD ectasia (3-5 mm) alone resulted in a nonsignificant increase in discrimination (eTable 3 in Supplement 1).

On NRI analysis (Table 3), adding age to cyst size–based stratification significantly improved overall classification of risk (NRI, 0.20; 95% CI, 0.03-0.37), with marked improvement among events (NRI, 0.58; 95% CI, 0.41-0.75). Adding MPD ectasia alone produced smaller, nonsignificant improvements in risk reclassification (Table 3).

**Table 3.** Net Reclassification Index (NRI) of Adding Age and/or Ductal Ectasia to Cyst Size–Based Risk Stratification

Variable	NRI (95% CI)		
	Overall	Event	Nonevent
Age ≥70 y	0.20 (0.03 to 0.37)	0.58 (0.41 to 0.75)	−0.38 (−0.39 to −0.36)
MPD ectasia of 3 to 5 mm	0.06 (−0.02 to 0.17)	0.10 (0.02 to 0.21)	−0.04 (−0.05 to −0.04)

Abbreviation: MPD, main pancreatic duct.

## Discussion

In this multisite cohort study, the incidence of pancreatic cancer among patients with low-risk PCLs was 1.89 cases per 1000 person-years, approximately 14-fold higher than the reported incidence in the general population (0.14 cases per 1000 person-years). Notably, 31.6% of cancers originated outside the cyst site, and 26.3% occurred more than 5 years after PCL detection. We found that larger PCL size, MPD ectasia, and older patient age were independent risk factors for pancreatic cancer development. Incorporating age into the cyst size–based model improved risk stratification of low-risk PCLs.

Prior studies have defined low-risk PCLs based on the absence of high-risk stigmata and worrisome imaging and clinical features.^9,10,11,12,13,14,15,37^ Although there is a variation in definition between guidelines, a substantial overlap exists, and we used the most recent consensus by Gonda et al.^8^ To date, the exact incidence of pancreatic cancer in patients with low-risk PCLs remains uncertain.^8^ In a meta-analysis that pooled data from 18 studies on patients with low-risk PCLs, the incidence of advanced neoplasia or cancer was 0.6% (95% CI, 0.2%-1.0%) over 5 years, which increased to 1.0% (95% CI, 0.6%-1.5%) beyond the 5 years of follow-up.^38^ These findings align closely with our study’s demonstrated incidence; however, pooled estimates from the meta-analysis showed substantial between-study heterogeneity (eg, *I*^2^ = 92%), limiting interpretability and direct translation of results into uniform surveillance recommendations.

Some studies have reported higher incidence, but they were often subject to selection bias by including patients with high-risk PCLs undergoing surgery.^39,40,41,42^ For instance, Nagai et al^39^ found that up to 61% of intraductal papillary mucinous neoplasms were associated with malignant neoplasm, but this estimate was likely biased due to the inclusion of only resected cases. Conversely, some studies have shown that the incidence of pancreatic cancer in patients with low-risk PCLs is similar to that among individuals without PCLs.^43,44^ Despite the overall low incidence of pancreatic cancer, our findings suggest that patients with low-risk PCLs may still have a 10-fold to 19-fold higher risk of pancreatic cancer compared with the general population.^35,36^ However, our cohort is not representative of the general population because our inclusion criteria required adequate follow-up imaging. More population-based cohort studies are needed to accurately identify the true risk of pancreatic cancer in patients with low-risk PCLs.

Considerable variation exists in guideline-recommended surveillance for low-risk PCLs.^9,10,11,12,13,14,15,37^ For instance, a newly detected 2.5-cm low-risk PCL warrants follow-up MRI in 1 year according to the 2015 American Gastroenterological Association Guideline,^14^ whereas the 2024 International Consensus Fukuoka Guidelines^12^ recommend 2 imaging studies—EUS, MRI, or CT—within 6 months. Similarly, recommendations for the duration of imaging follow-up range from 5 years to lifelong.^14,15^ Most guidelines stratify surveillance based on cyst size, while some guidelines incorporate patient age and comorbidities to refine risk assessment.^9,10,11,12,13,14,15,37^ These guidelines are largely based on expert opinion due to the lack of robust longitudinal data.^9,10,11,12,13,14,15,37^

A key objective in PCL management is to refine surveillance strategies, ensuring timely detection of high-risk lesions. To date, the optimal strategy for risk stratification in low-risk PCL surveillance remains undefined.^8^ Our study helps address this evidence gap by demonstrating that the maximal cyst size is one of the factors independently associated with pancreatic cancer development in patients with low-risk PCLs. Furthermore, incorporating age into cyst size–based surveillance is associated with meaningfully improved risk stratification. While 23.7% of pancreatic cancers were diagnosed within 1 year and 50.0% were diagnosed between 1 and 5 years after the index examination, 26.3% developed 5 years after detection of low-risk PCLs, supporting continued longer-term follow-up in these patients.

Additionally, our data revealed that cancer developing from PCLs larger than 1 cm more often originated at the cyst site, whereas cancer from cysts smaller than 1 cm tended to originate elsewhere in the pancreas. Finally, the observation that pancreatic cancer developed outside the index cyst in 31.6% of patients may reflect the field defect phenomenon.^8,45,46^ In this context, diffuse molecular and genetic alterations within the pancreatic ductal epithelium may predispose to multifocal neoplastic transformation rather than malignant progression of the index cyst alone, supporting surveillance strategies that emphasize evaluation of the entire pancreas rather than a cystic lesion alone.

### Limitations

This study has several limitations. First, although the cohort size was substantial, an even larger sample size could have improved statistical power, particularly given the low incidence of pancreatic cancer. However, assembling a larger dataset with long-term follow-up remains challenging; a meta-analysis of prior studies on patients with PCL included only 10 345 cases.^38^ Second, the retrospective design introduced variability in follow-up intervals and durations. Additionally, lacking adequate follow-up may have led to an underestimation or overestimation of cancer incidence. Despite this variability, our findings reflect clinical practice. Third, this study relied on imaging-based diagnosis without histopathologic confirmation, an inherent limitation as most low-risk PCLs are managed without surgery. However, given the imaging characteristics and communication with MPD, we presumed that a large portion of the included cases were intraductal papillary mucinous neoplasms. Moreover, although positive findings from EUS-guided FNA were used to define pancreatic cancer in the current cohort, negative results from EUS-guided FNA were not systematically analyzed, as most patients underwent longitudinal surveillance with cross-sectional imaging, which served as the primary basis for censoring.

Fourth, laboratory biomarkers such as carbohydrate antigen 19-9, hemoglobin A_1c_, and serum glucose were not available for most included patients. Fifth, this study was not designed to develop or validate a prediction model, and the limited number of events precluded reliable model development or data splitting. Discrimination metrics were therefore presented as exploratory analyses and should be interpreted as descriptive measures of risk stratification rather than predictive performance. Sixth, identification of clinically meaningful thresholds (eg, for age) would require decision-analytic approaches incorporating clinical trade-offs and stakeholder input, which were beyond the scope of this study. Seventh, patients who developed pancreatic neuroendocrine tumors were excluded because they represent a biologically distinct entity with different management pathways. Eighth, the 95% CI for MPD ectasia was wide, reflecting the limited number of events in this subgroup. Ninth, the cohort was derived from a single, large health care system and consisted predominantly of White patients, which may limit the generalizability of these findings. Finally, our analysis focused on baseline characteristics, whereas future studies incorporating longitudinal changes in cyst features may further improve risk assessment.

## Conclusions

In this retrospective multisite cohort study of patients with low-risk PCLs, larger cyst size, MPD ectasia, and older age were independently associated with pancreatic cancer development. Incorporating age into cyst size–based surveillance strategies was associated with improved risk stratification. Given that 26.3% of pancreatic cancers were diagnosed beyond 5 years of follow-up, longer than a 5-year follow-up of low-risk PCLs may be needed to reduce missed or delayed diagnosis.

## Notes

Supplement 1. **eTable 1.** Missing Data Summary **eTable 2.** Distribution of Pancreatic Cancer Cases by Baseline Cyst Size and Time to Diagnosis Among Patients With Low-Risk Pancreatic Cystic Lesions **eTable 3.** Harrell’s C-statistic: Adding Age and/or Ductal Ectasia to Cyst Size-based Risk Stratification
eMethods.

Supplement 2.
Data Sharing Statement
