# Supplementary material for: Pancreatic Cancer Risk in Patients With Low-Risk Cystic Lesions
Source: JAMA Netw Open. 2026 May 20;9(5):e2613808. doi: 10.1001/jamanetworkopen.2026.13808 (PMC13191383; doi:10.1001/jamanetworkopen.2026.13808)
Supplement: Supplement 1. — eTable 1. Missing Data Summary eTable 2. Distribution of Pancreatic Cancer Cases by Baseline Cyst Size and Time to Diagnosis Among Patients With Low-Risk Pancreatic Cystic Lesions eTable 3. Harrell’s C-statistic: Adding Age and/or Ductal Ectasia to Cyst Size-based Risk Stratification eMethods. [file jamanetwopen-e2613808-s001.pdf]

Supplemental Online Content

Haj Mirzaian A, Abbasi N, Kambadakone AR, et al. Pancreatic cancer risk in patients with low-risk cystic lesions. *JAMA Netw Open*. 2026;9(5):e2613808. doi:10.1001/jamanetworkopen.2026.13808

eTable 1. Missing Data Summary

eTable 2. Distribution of Pancreatic Cancer Cases by Baseline Cyst Size and Time to Diagnosis Among Patients With Low-Risk Pancreatic Cystic Lesions

eTable 3. Harrell's C-statistic: Adding Age and/or Ductal Ectasia to Cyst Size-based Risk Stratification

eMethods

This supplemental material has been provided by the authors to give readers additional information about their work.

© 2026 Haj Mirzaian A et al. *JAMA Network Open*

eTable 1. Missing Data Summary

Variable	% Missing
Age, years	0.02%
Sex	0.02%
PCL Size	5.72%
Main Pancreatic Duct Ectasia	0.00%
Modality, MRI vs. CT	0.20%
Race	3.50%
Diabetes	19.66%
Obesity/Overweight	19.66%
Family History of Pancreatic Cancer	0.00%
Ever Smoked	3.61%
Active Smoker	0.00%
Amount of Alcohol Use, mean units per week	0.00%
PCL location	9.98%
Multiple PCLs	0.00%
Imaging Features of Chronic Pancreatitis	0.00%
Pancreatic Divisum	0.00%
Suspected Pseudocyst	0.00%
Suspected Collection	0.00%
Suspected Serous Cystadenoma	0.00%
Average Missing %	2.71%

PCL= Pancreatic Cystic Lesion

© 2026 Haj Mirzaian A et al. *JAMA Network Open*

eTable 2. Distribution of Pancreatic Cancer Cases by Baseline Cyst Size and Time to Diagnosis Among Patients with Low-Risk Pancreatic Cystic Lesions

PCL size	No. Total	No. cancer	% cancer	Time to diagnosis of pancreatic cancer (year) #		
				<1 y	1-5 y	>5 y
**<1 cm**	4188	18	0.4%	4 (1)	8 (4)	6 (3)
**1 – <2 cm**	1261	11	0.9%	3 (2)	8 (8)	0
**2 – <3 cm**	268	9	3.4%	2 (2)	3 (2)	4 (4)

Values are presented as the number of pancreatic cancer cases, with the number of cancers arising from the same PCL site (rather than other locations in the pancreas) shown in parentheses.

Baseline PCL size refers to the maximum diameter of the largest cyst on the index imaging examination. Time to diagnosis indicates the interval between baseline cyst detection and pancreatic cancer diagnosis, categorized as <1 year, 1 to 5 years, and >5 years. Numbers in parentheses reflect cancers that originated at the anatomical location of the PCL; remaining cases arose elsewhere in the pancreas.

© 2026 Haj Mirzaian A et al. *JAMA Network Open*

eTable 3. Harrell's C-statistic: Adding Age and/or Ductal Ectasia to Cyst Size-based Risk Stratification

	**C-statistic (95% CI)**	***P*-value*#**
Cyst Size (≥2 cm)		
Alone	0.57 (0.51 to 0.64)	
Adding Age (≥70 years)	0.71 (0.64 to 0.78)	**<.001**
Adding MPD Ectasia (3 to 5 mm)	0.61 (0.54 to 0.71)	.16

# Compared to Cyst Size–based Risk Stratification Model (Alone)

* Bold values represent p-value of <0.05

MPD= main pancreatic duct

© 2026 Haj Mirzaian A et al. *JAMA Network Open*

eMethods

The proportional hazards assumption was evaluated using Schoenfeld residuals and inspection of log-minus-log survival plots. No violations of the proportional hazards assumption were observed (all $P>0.05$).

Because part of the follow-up period overlapped with the COVID-19 pandemic, we performed additional analysis by including a COVID-19 period indicator (March 2020 onward) in the multivariable cause-specific Cox model. Adjustment for the COVID-19 period was not associated with pancreatic cancer risk (HR, 1.01; 95% CI, 0.75–1.35; $P=0.97$).

© 2026 Haj Mirzaian A et al. *JAMA Network Open*
