# Supplementary material for: Pancreatic Cancer Risk in Patients With Low-Risk Cystic Lesions
Source: JAMA Netw Open. 2026 May 20;9(5):e2613808. doi: 10.1001/jamanetworkopen.2026.13808 (PMC13191383; doi:10.1001/jamanetworkopen.2026.13808)
Supplement: Supplement 2. — Data Sharing Statement [file jamanetwopen-e2613808-s002.pdf]

# Data Sharing Statement

Haj Mirzaian. Pancreatic Cancer Risk in Patients With Low-Risk Cystic Lesions. *JAMA Netw Open*. Published May 20, 2026. doi:10.1001/jamanetworkopen.2026.13808

**Data**
**Data available:** No
